# Assessing a single SNP located at *TERT/CLPTM1L* multi-cancer risk region as a genetic modifier for risk of pancreatic cancer and melanoma in Dutch *CDKN2A* mutation carriers

**DOI:** 10.1007/s10689-019-00137-5

**Published:** 2019-06-15

**Authors:** E. Christodoulou, M. Visser, T. P. Potjer, N. van der Stoep, M. Rodríguez-Girondo, R. van Doorn, N. Gruis

**Affiliations:** 1 Department of Dermatology, Leiden University Medical Center (LUMC), Albinusdreef 2, 2333 ZA Leiden, The Netherlands; 2 Department of Clinical Genetics, LUMC, Albinusdreef 2, 2333 ZA Leiden, The Netherlands; 3 Section of Medical Statistics, Department of Biomedical Data Sciences, LUMC, Einthovenweg 20, 2333 ZC Leiden, The Netherlands

**Keywords:** *CDKN2A*, *p16-Leiden*, Pancreatic cancer, Melanoma, Genetic modifiers, SNP

## Abstract

Carriers of pathogenic variants in *CDKN2A* have a 70% life-time risk of developing melanoma and 15–20% risk of developing pancreatic cancer (PC). In the Netherlands, a 19-bp deletion in exon 2 of *CDKN2A* (*p16-Leiden* mutation) accounts for most hereditary melanoma cases. Clinical experience suggests variability in occurrence of melanoma and PC in *p16-Leiden* families. Thereby, the risk of developing cancer could be modified by both environmental and genetic contributors, suggesting that identification of genetic modifiers could improve patients’ surveillance. In a recent genome-wide association study (GWAS), rs36115365-C was found to significantly modify risk of PC and melanoma in the European population. This SNP is located on chr5p15.33 and has allele-specific regulatory activities on *TERT* expression. Herein, we investigated the modifying capacities of rs36115365-C on PC and melanoma in a cohort of 283 *p16-Leiden* carriers including 29 diagnosed with PC, 171 diagnosed with melanoma, 21 diagnosed with both PC and melanoma and 62 with neither PC nor melanoma. In contrast to previously reported findings, we did not find a significant association of PC risk with risk variant presence as determined by Generalized Estimating Equations (GEE) modelling. Interestingly, carrier-ship of the risk variant had a significant protective effect for melanoma (OR − 0.703 [95% CI − 1.201 to − 0.205], p = 0.006); however, the observed association was no longer significant after exclusion of probands to assess possible influence of ascertainment. Collectively, genetic modifiers for the prediction of PC and melanoma risk in *p16-Leiden* carriers remain to be determined.

## Introduction

*CDKN2A* is the major high-risk susceptibility gene identified thus far for familial melanoma [1]. In the Netherlands, the most common cause of familial melanoma is a *CDKN2A* founder mutation which is a deletion of 19 bp in exon 2 (c.225_243del, p.(A76Cfs*64); RefSeq NM_000077.4), also known as the *p16-Leiden* mutation resulting in inactivation of tumor-suppressive properties of p16(INK4a) [2]. Carriers have a lifetime risk of 70% to develop melanoma [3], and a life-time risk of 15–20% to develop pancreatic cancer (PC) [4–6].

PC is a highly aggressive cancer subtype with very poor prognosis resulting in a 5-year survival rate of < 5% [7]. It is therefore one of the leading causes of cancer-related deaths worldwide [8], suggesting there is much to gain by early detection of PC at a stage when surgical removal is still curative [9]. Carriers of the *p16-Leiden* mutation are advised to undergo screening yearly for PC using MRI from the age of 45 [6].

Clinical studies of *p16-Leiden* mutated families, have shown variability in occurrence of melanoma and PC among families suggesting contribution of modifying factors to cancer risk [4]. For example, genetic risk factors such as *MC1R*, were found to modify risk of developing melanoma in *p16-Leiden* positive families significantly [10, 11]. Therefore, the variable occurrence of PC in those families might also be explained by modifying genetic risk factors other than the *p16-Leiden* mutation. Determination of those factors would allow for a better identification of patients at increased risk that might benefit from personalized clinical management.

In an attempt to identify genetic factors that modulate the risk of pancreatic cancer in *p16-Leiden* carriers, Potjer et al. analyzed seven SNPs associated with PC risk in the general population in this cohort of carriers and found no significant association [12]. Recently a risk variant, rs36115365-C, was identified to be significantly correlated with PC risk in the European population [13]. This SNP is located at a multi-cancer risk locus on chr5p15.33 and was found to have allele-specific regulatory activities on *TERT* expression, mutations of which have been associated with melanoma risk [14]. These data suggest that variation within rs36115365 (G,C) could contribute to cancer development. Indeed, carriers of the minor C-allele are at increased risk of pancreatic cancer (RR = 1.2). Remarkably at the same time, carriers of this C-allele are at diminished risk of developing melanoma [13]. Several risk variants have been reported to be associated with a small but important protective effect against melanoma in sporadic melanoma such as variants in *GSTM1* and *GSTT1* [15] and polymorphisms in the Vitamin D receptor gene [16]. These findings collectively suggest that identification of genetic modifiers in *p16-Leiden* carriers could be used to estimate the risk of developing PC and melanoma more accurately. This study therefore, investigates and verifies the risk impact of the reported SNP variant, rs36115365-C in a Dutch *p16-Leiden* positive patient cohort.

## Methods

### Cohort description

The study population included only confirmed *p16-Leiden* carriers of which DNA samples were available from the Laboratory for Diagnostic Genome Analysis (LDGA) of Leiden University Medical Center (LUMC). Subject-specific clinical information was collected between 1998 and the 1st of January, 2015. Cases of PC were individuals carrying the *p16-Leiden* mutation who were diagnosed with primary exocrine PC. Similarly, cases of melanoma were *p16-Leiden* carriers who were diagnosed with cutaneous (multiple) melanoma. Detailed medical record data on the study population has been reported previously [12]. Approval of this study was obtained from the ethics committee of Leiden University Medical Center (LUMC #P14.148) [12].

In total, 419 *p16-Leiden* carriers were available for inclusion in the current study. The comparative analysis was formulated by filtering for carriers diagnosed with PC, carriers diagnosed with melanoma, and a group who did neither develop PC nor melanoma but were older than 55 years of age. Subsequently, a master cohort of 283 *p16-Leiden* carriers from a total of 121 *p16-Leiden* families were included. These consisted of 29 carriers with PC (median age 47 years), 171 with melanoma (median age 60), 21 with both PC and melanoma (median age 60) and 62 with neither PC nor melanoma (median age 71) (Table 1).

### Genotyping and statistical analysis

Genotyping analysis was carried out using the rhAmp-SNP Genotyping Assay (Integrated DNA Technologies (IDT), Leuven, Belgium). Bi-allelic discrimination was achieved by incorporation of two forward primers specifically targeting the allele of interest (rs36115365-C). The genotyping procedure was performed according to manufacturer’s instructions. The reference allele was labelled with FAM reporter dye and the alternate allele with Yakima Yellow (YY) reporter dye which were both detected on CFX384 Touch Real-Time PCR Detection System (Bio-Rad, Veenendaal, The Netherlands) with excitation sources and emission filters for respective wavelengths. The minor allele frequency (MAF) was calculated based on Hardy–Weinberg law. For statistical analysis, a generalized linear model with logit link was used to assess the association between alternate-allele presence and risk for PC and melanoma development. The binary dependent variable was either PC or melanoma and SNP variant was the explanatory indicator variable. Regression coefficients and 95% Confidence Intervals (CI) were calculated. *P-*values of < 0.05 were considered statistically significant.

Due to nature of this family-based study, individuals are not independent, they are clustered. In order to account for this feature, a Generalized Estimating Equations (GEE) procedure was used to fit the model. The GEE procedure allows to deal with clustered data. Since the specific correlation structure of this data is difficult to estimate due to the small sample size, an independence working correlation structure was assumed. To avoid the impact of possible misspecification of the model in the confidence intervals and p-values, robust estimates of the standard errors were obtained using a sandwich estimator [17]. All statistical analyses were performed using IBM SPSS Statistics 23.

## Results

The risk impact of rs36115365-C on PC was calculated by comparing a total of 50 *p16-Leiden* carriers who developed PC (median age 50) to 143 controls (median age 74) consisting of 62 carriers who did not develop PC and 81 carriers who developed melanoma but were older than 55 years of age (sub-cohort characteristics, Table 2). Similarly, the risk impact of rs36115365-C on melanoma was calculated by comparing a total of 192 *p16-Leiden* carriers who developed melanoma (median age 60) to 73 controls (median age 71), consisting of 62 carriers who did not develop melanoma and 11 carriers who developed PC but were older than 55 years of age (sub-cohort characteristics, Table 3). The latter group in both analyses was treated as a control since it consisted of *p16-Leiden* carriers older than 55 years of age with a subsequent reduced risk of developing melanoma or pancreatic carcinoma in the future.

MAF of the risk variant rs36115365-C for different comparison groups, Beta values and 95% CI were calculated (Table 4). No significant association was found for risk variant presence and PC risk (Table 4). Interestingly, a significant negative association was observed for risk variant carriers and melanoma development suggesting a protective effect of rs36115365-C for melanoma in *p16-Leiden* carriers (OR − 0.703, 95% CI (− 1.201, − 0.205), p-value = 0.006) (Table 4). To assess possible influence of ascertainment in the sample cohort, the association with melanoma was further explored by excluding probands. This resulted in a decreased cohort size of 69 families consisting of 158 *p16-Leiden* carriers, 85 of whom had developed melanoma. The statistical significant association did not remain in that case for risk variant carriers (GEE model − 0.453, 95% CI (− 1.051, 0.145), p-value = 0.138).

**Table 1:** Master cohort characteristics of *p16-Leiden* carriers

	*p16-Leiden* carriers (N = 283)			
	Pancreatic cancer (N = 29)*	Melanoma (N = 171)*	Pancreatic cancer and melanoma (N = 21)	Non-melanoma, non-pancreatic cancer (N = 62)*
Median age (years)	47 (21–72)	60 (27–93)	60 (42–78)	71 (55–86)
Gender (M:F)	9:20	71:100	8:13	26:36
Multiple melanoma	–	71/171 (42%)	6/21 (29%)	–
Patients diagnosed with other cancer	3	33	6	24

*p16-Leiden carriers who developed either pancreatic cancer or melanoma or neither of the two and were older than 55 years of age served as controls in comparative analysis, see Tables 2 and 3

**Table 2:** Sub-cohort characteristics of *p16-Leiden* carriers with/without pancreatic cancer

	Pancreatic cancer cases (N = 50)	Non-Pancreatic cancer controls (N = 143)
Median age (years)	50 (21–78)	74 (55–93)
Gender (M/F)	17/33	60/83
Medical history of melanoma	21	81
Multiple melanoma	6/21 (29%)	37/81 (46%)
Patients diagnosed with other cancer	9	51

**Table 3:** Sub-cohort characteristics of *p16-Leiden* carriers with/without melanoma

	Melanoma cases (N = 192)	Without melanoma controls (N = 73)
Median age (years)	60 (27–93)	71 (55–86)
Gender (M/F)	79/113	28/45
Medical history of pancreatic cancer	21	11
Multiple melanoma	77/192 (40%)	–
Patients diagnosed with other cancer	54	24

**Table 4:** Association of rs36115365-C presence with PC and melanoma in *p16 –Leiden* carriers

Pancreatic cancer	MAF rs36115365 (G,C)		Allelic OR	95% CI		p-value
	Cases	Controls				
Pancreatic cancer	0.23	0.23	− 0.027	− 0.804	0.750	0.946
Melanoma	0.21	0.29	− 0.703	− 1.201	− 0.205	0.006

## Discussion

Identification of genetic modifiers for PC and melanoma risk in *p16-Leiden* carriers could possibly explain the variability of cancer occurrence within *p16-Leiden* positive families and ultimately favor individualized surveillance and clinical management of those patients [4, 12]. Herein, we sought to estimate the risk of developing PC and melanoma more accurately in carriers of the pathogenic variant *p16-Leiden*. This was tested by determining whether a previously published associated risk variant for PC and melanoma, rs36115365-C [13], could explain modified risk in a homogeneous population of *p16-Leiden* carriers.

The MAF of rs36115365-C in the general European (Non-Finnish) population is 0.18 [18] and in the Netherlands specifically, it is 0.20 [19] indicating a common variant with high chances of detection. In this case, the second most-common allele (C) was detected with MAFs ranging from 0.21 to 0.29 in 283 *p16-Leiden* carriers, slightly higher than in the general population. A limitation of this study however is the small cohort size that could limit possibilities of detecting statistical associations. Moreover, selecting subjects older than 55 years of age not only reduced the control group size but also the possibility of developing PC or melanoma in the future was not fully excluded. A GEE statistical procedure was applied as it is appropriated for studying family based associations [17, 20]. There was no significant association between rs36115365-C presence and PC risk in *p16-Leiden* carriers.

Several efforts in scientific literature focused on identifying genetic modifiers of PC risk in *CDKN2A*-mutation carriers. Yang et al., applied Whole Exome Sequencing (WES) in 66 PC patients with/without a *CDKN2A* mutation [21]. The combined data from five research groups, including 13 pancreatic *CDKN2A* mutated (*p16-Leiden*) cases from the Netherlands identified 35 variants in PC-related genes. Nominally significant associations were obtained for mismatch repair genes (*MLH1, MSH2, MSH6, PMS2*) in all PC patients, however, variants in *ATM, CPA1*, and *PMS2* were only observed in *CDKN2A* wild-type PC patients. Further, nine *CDKN2A* mutated and four *CDKN2A* wild-type PC patients had rare potentially deleterious variants in multiple PC-related genes. These results, therefore, suggest that a subset of PC patients may have increased risk because of germline mutations in multiple PC-related genes [21]. Nonetheless, *p16-Leiden* carriers described in the study by Yang were not included in the current study. In addition, the same group showed that sequencing analysis of *PALB2*, another high susceptibility gene for PC, did not reveal any deleterious mutations in PC patients from *CDKN2A*-mutated families [22]. Potjer et al. who studied the same cohort of *p16-Leiden* carriers as in the current study, did not identify an association of seven PC-associated SNPs with PC risk [12]. Therefore, consideration of other genetic modifiers yet unknown could be an additional explanation of the variability in occurrence of PC within *p16-Leiden* families. Moreover, epidemiological studies suggest that non-genetic factors may also contribute to PC development specifically in *p16-Leiden* carriers, with the most significant one being smoking [23] as well as alcohol use and obesity in the general population [24]. Collectively these data suggest that rs36115365-C risk variant could not be used to estimate the risk of PC in *p16-Leiden* mutation carriers more accurately unlike in the European population published previously [13].

Modifier genes for melanoma have been well described in literature for *CDKN2A* mutation carriers [10, 11, 25, 26]. The variant rs36115365-C, previously published to be negatively correlated with melanoma risk [13] had a significantly negative association with melanoma development in this study. This effect did not remain however, when excluding probands from the analysis suggesting that ascertainment of melanoma cases influenced the results.

## Conclusion

Here, no significant association was found between rs36115365-C presence and risk of PC development in *p16-Leiden* carriers in contrast to previous published literature in the European population. Reversely, a statistically significant protective effect was determined for melanoma risk in the same cohort of *p16-Leiden* carriers, an effect that lost significance when excluding melanoma probands. Collectively, genotyping and statistical data suggest that genetic modifiers for the prediction of PC and melanoma in *p16-Leiden* carriers remain to be determined.
